# Enhanced immunotherapy by combining a vaccine with a novel murine GITR ligand fusion protein

**DOI:** 10.18632/oncotarget.20703

**Published:** 2017-09-07

**Authors:** Y. Maurice Morillon, Scott A. Hammond, Nicholas M. Durham, Jeffrey Schlom, John W. Greiner

**Affiliations:** ^1^ Laboratory of Tumor Immunology and Biology, Center for Cancer Research, National Cancer Institute, National Institutes of Health, Bethesda, MD, USA; ^2^ MedImmune LLC, Gaithersburg, MD, USA

**Keywords:** poxvirus vaccines, rMVA/rF-CEA-TRICOM, mGITRL-FP, CD4^+^FoxP3^+^ regulatory T cells, immunotherapy

## Abstract

Immunotherapy was significantly enhanced in a murine tumor model by combining a vaccine with a fusion protein designed to target the glucocorticoid-induced tumor necrosis factor (TNF) receptor related gene (GITR) on the surface of T cells. The recombinant poxvirus-based vaccine platform included Modified Vaccinia virus Ankara (rMVA) and fowlpox (rF) vectors as the driver immunogens both engineered to express the human carcinoembryonic antigen (CEA) and three murine costimulatory molecules B7.1, ICAM-1, LFA-3 (designated TRICOM). In previous studies, mice expressing human *CEA* as a transgene (CEA.Tg mice) vaccinated with rMVA/rF-CEA-TRICOM overcame CEA immune tolerance by inducing anti-CEA‒specific immunity and regression of CEA-expressing tumors. The murine GITR ligand fusion protein (mGITRL-FP) consisted of a mouse IgG2a Fc region, a yeast-derived coiled GCN4 pII and the extracellular GITR-binding domain of murine GITR ligand. The design maximized valency and the potential to agonize the GITR receptor. Combined treatment of the vaccine and mGITRL-FP mediated a more robust tumor regression, leading to sustained improvement in overall survival. The enhanced immunotherapeutic effect was linked to the generation of a strong CD8^+^ T cell antitumor immune response. A treatment schedule with mGITRL-FP administered prior to the priming rMVA-CEA-TRICOM vaccination was of paramount importance. The mechanism of action for the enhanced antitumor effects resided in the depletion of immune cells, particularly FoxP3^+^ regulatory T cells, that express high GITR levels following activation. The results provide evidence that targeting GITR with mGITRL-FP in concert with a cancer vaccine represents a potential novel approach to more effective immunotherapy.

## INTRODUCTION

Glucocorticoid-induced tumor necrosis factor (TNF) receptor related gene or “GITR,” also known as TNF receptor superfamily member 18 (TNFRSF18), was listed by the National Cancer Institute as among the most promising immunotherapy agents for cancer [1]. GITR is expressed on innate and adaptive components of the immune system including CD4^+^, CD8^+^ T cells, natural killer (NK) cells, B cells, macrophages and dendritic cells (DCs), and functions as a key regulator of inflammatory and immune responses [2-4]. GITR expression on resting CD4^+^FoxP3^-^ and CD8^+^ T cells is low, but upon T cell receptor (TCR) engagement, it is upregulated and acts as a costimulatory molecule enhancing T cell proliferation and cytokine production [5, 6]. Cross-linking of GITR reduces TCR-induced apoptosis [7] and improves T cell survival by maintaining responsiveness through signaling of multiple protein kinases (i.e., MAPK, ERKs, JNKs, etc.) and nuclear factor kB (NF-kB) pathways [8-10]. CD4^+^FoxP3^+^ regulatory T cells (T_reg_) constitutively express high levels of GITR and GITR ligation breaks self-tolerance and abrogates T cell suppression by T_reg_, thus providing the rationale to target GITR for cancer immunotherapy [7, 10]. Treatment with an agonist anti-GITR antibody (DTA-1) leads to tumor regression, improved T cell effector function and induced long-lasting immune memory that protected against tumor rechallenge [11-14]. Multiple mechanisms seem to work in concert that ultimately manifest the antitumor effects of targeting GITR. They include: (a) impairment of intratumoral T_reg_ expression of FoxP3, resulting in a loss of T_reg_ cell lineage stability and abrogation of intratumoral T_reg_ suppressive function [15], (b) downregulation of exhaustion markers for CD8^+^ intratumoral T cells increasing their CTL function [16] and (c) generation of high-avidity CTL responses to tumor-associated antigens [17]. In more recent studies, targeting GITR, particularly on T_regs_, using the anti-GITR monoclonal antibody, clone DTA-1, has led to T_reg_ depletion, which is linked to FcγR function [18, 19].

A platform of recombinant poxvirus-based vaccines has been developed that include Modified Vaccinia virus Ankara (rMVA) as a prime and fowlpox (rF) as boosters, both expressing as transgenes, human carcinoembryonic antigen (CEA) and three costimulatory molecules B7.1, ICAM-1, LFA-3 (designated TRICOM), thus termed rMVA/rF-CEA-TRICOM [20, 21]. CEA, a *M*_*r*_180,000-200,000 glycoprotein, like other oncofetal antigens, is overexpressed by a high percentage of human colorectal cancer, colonic polyps and other adenocarcinomas [22-24], and to a lesser extent by normal human colonic mucosa. Enhanced expression levels of those self-antigens on tumors is believed to provide an opportunity to develop antigen-specific vaccines capable of breaking immune tolerance, thus generating anti-CEA host immune responses. Using preclinical mouse models expressing the complete human *CEA* gene as a transgene [25], several different poxvirus CEA-directed vaccines were shown to overcome CEA immune tolerance by inducing anti-CEA specific immunity, which, in turn, correlated with the regression of CEA-expressing tumors [26-29]. Despite those successes, there remains a need to improve the overall efficacy of those and most other therapeutic cancer vaccines.

A previous study described the preclinical characterization of a multimeric mouse GITR ligand fusion protein designed to maximize valency and the potential to agonize the GITR receptor [30]. The murine GITR ligand fusion protein (mGITRL-FP) consisted of an IgG2a Fc domain, a yeast-derived coiled GCN4 pII and the extracellular GITR-binding domain of murine GITR ligand. The mGITRL-FP and DTA-1 induced NF-κB signaling in a GITR-dependent NF-κB reporter gene cell assay. However, the EC_50_ for the mGITRL-FP was 0.05 nM, nearly 50 times more potent than DTA-1 whose EC_50_ was 2.31 nM [30]. Administration of mGITRL-FP reduced the growth of CT26 s.c. tumors in a dose-dependent manner which correlated with (a) enhanced expression of proliferative/activation markers on peripheral T cells and (b) reduction of intratumoral T_regs_. Increasing the T_effector_/T_reg_ ratio at the tumor microenvironment by targeting GITR with the mGITRL-FP could be considered an immune adjuvant and, thus, may be an effective approach to enhance the antitumor efficacy of a cancer vaccine. That hypothesis was tested in the current study by combining the rMVA/rF-CEA-TRICOM vaccine platform with the murine multimeric GITR ligand fusion protein (mGITRL-FP) in CEA transgenic (CEA.Tg) mice bearing CEA-expressing tumors. Both the rMVA/rF-CEA-TRICOM vaccine and mGITRL-FP induced measurable tumor regression when administered as monotherapies. By combining those two immune-based therapies, antitumor effects were significantly enhanced resulting in complete tumor regression, significant prolongation of tumor-free survival and the generation of protective immune memory. These current findings provide the rationale for potential clinical studies combining these two immunotherapeutic platforms.

## RESULTS

### Dosing/timing schedules for the combined rMVA/rF-CEA-TRICOM and mGITRL-FP treatment

**Figure 1:**
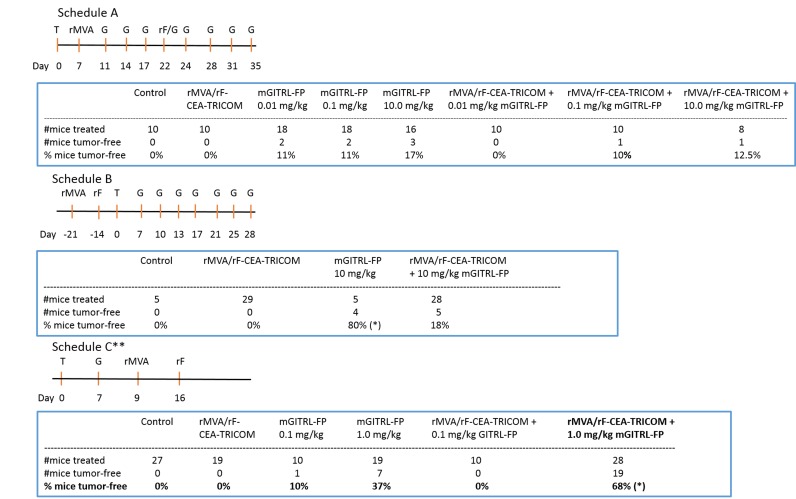
Anti-tumor efficacy is dependent on dosage and timing of mGITRL-FP combined with rMVA/rF-CEA-TRICOM vaccination Schedule A-C summarizes the time points (Day) of tumor (T) inoculation, mGITRL-FP (G) administration, and recombinant Modified Vaccinia Ankara (rMVA-CEA-TRICOM: rMVA) and recombinant fowlpox (rF-CEA-TRICOM: rF) vaccinations. ** indicates the treatment schedule used for subsequent studies. *P ≤ 0.05; Log-rank (Mantel-Cox) test; comparison of mGITRL-FP 10 mg/kg vs rMVA/rF-CEA-TRICOM + mGITRL-FP 10 mg/kg (Schedule B); comparison of mGITRL-FP 1.0 mg/kg vs rMVA/rF-CEA-TRICOM + mGITRL-FP 1.0 mg/kg (Schedule C).

**Figure 2:**
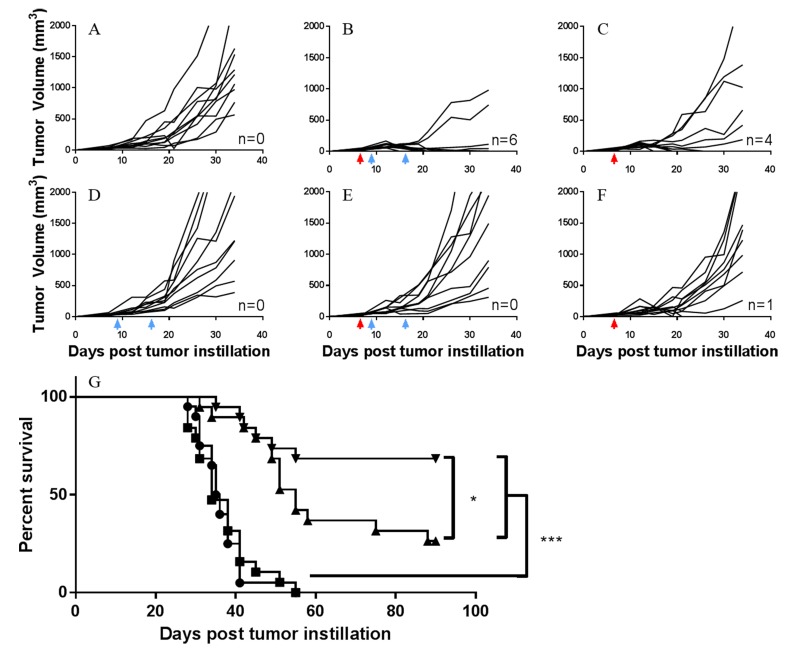
Combination of rMVA/rF-CEA-TRICOM with mGITRL-FP induces complete regression of CEA-expressing tumors in CEA.Tg mice CEA.Tg mice (*n* = 9) bearing MC32A s.c. tumors were treated with either 0.1 or 1.0 mg/kg mGITRL-FP (red arrows) on day 7 post-tumor injection, followed on days 9 and 16 with rMVA/rF-CEA-TRICOM immunizations, respectively (blue arrows). Individual tumor growth curves are shown for **A.** control, untreated, **B.** vaccine + mGITRL-FP (1.0 mg/kg), **C.** mGITRL-FP (1.0 mg/kg), **D.** vaccine alone, **E.** vaccine + mGITRL-FP (0.1 mg/kg), and **F.** mGITRL-FP (0.1 mg/kg)-treated CEA.Tg mice; *n* values denote number of CEA.Tg mice cured of MC32A tumors. Data are from a representative experiment that was repeated twice with similar results. **G.** Survival curve of MC32A tumor bearing CEA.Tg mice of untreated (circles) or treated with either vaccine (squares) or mGITRL-FP (1.0 mg/kg) (triangles) alone or in combination (inverted triangles) (*n* = 19; Log-rank (Mantel-Cox) test; *P ≤ 0.05, vaccine + mGITRL-FP vs. mGITRL-FP alone; ***P ≤ 0.001, vaccine + mGITRL-FP or mGITRL-FP alone vs. vaccine alone).

Initial studies examined different dosing and treatment schedules to optimize the combined immunotherapeutic effects of the rMVA/rF-CEA-TRICOM vaccine and mGITRL-FP in CEA.Tg mice bearing MC32A tumors (Figure 1). In several experiments, mGITRL-FP was co-administered, at doses ranging from 0.01-10 mg/kg, in combination with the vaccine. No additional antitumor effects were observed when the vaccine was administered prior to mGITRL-FP and compared with either monotherapy (see Figure 1, Schedule A). In a subsequent study, CEA.Tg mice were initially vaccinated and 14 days after the booster vaccination (i.e., rF-CEA-TRICOM) challenged with MC32A tumor cells with mGITRL-FP treatment (10 mg/kg; Q2W) beginning 7 days later (see Figure 1, Schedule B). The vaccine alone slowed MC32A tumor growth, while treatment with 10 mg/kg mGITRL-FP as a monotherapy induced tumor regression (Figure 1, Schedule B). Tumor regression following the administration of 10 mg/kg GITRL-FP as a monotherapy was observed only in those CEA.Tg mice with low tumor volume on day 7 post-tumor inoculation (see Figure 1, Schedule A vs. B). However, combining the two immunotherapeutics resulted in a loss of antitumor efficacy when compared with MC32A‒tumor bearing CEA.Tg mice treated with mGITRL-FP alone (Figure 1, Schedule B). Significant improvements in the antitumor efficacy of the vaccine occurred when MC32A tumor bearing CEA.Tg mice received a single i.p. injection of 1.0 mg/kg mGITRL-FP 2 days prior to the priming vaccine followed on day 16 with the rF-CEA-TRICOM booster vaccination (see Figure 1, Schedule C; Figure 2A and 2B). Combined results from two independent studies revealed complete MC32A tumor regression in 68% (19 of 28) (Figure 1; *P ≤ 0.05) of the CEA.Tg mice. Administration of mGITRL-FP alone at 1.0 mg/kg did induce MC32A tumor regression in CEA.Tg mice, but the extent of tumor regression was not significant when compared with control-treated CEA.Tg mice (Figure 2C). CEA.Tg mice bearing MC32A tumors and treated with the vaccine alone (Figure 2D) or combined with a lower dose of mGITRL-FP (0.1 mg/kg) (Figure 2E) resulted in no significant changes in MC32A tumor growth. Likewise, treatment of CEA.Tg mice with a lower dose of mGITRL-FP (0.1 mg/kg) alone resulted in no significant change in MC32A tumor growth (Figure 2F). Using Schedule C (Figure 1), CEA.Tg mice treated with vaccine and 1.0 mg/kg mGITRL-FP had a significant improvement in overall tumor-free survival (***P ≤ 0.001, Figure 2G). These findings provide the framework to examine the possible consequences of mGITR-FP targeting GITR on immune cell subsets and the impact on the antitumor efficacy of the vaccine. All subsequent studies were performed using Schedule C (Figure 1) in which mGITRL-FP was administered 2 days prior to the priming vaccine followed by the booster vaccination.

### Targeting and depletion potential of mGITRL-FP includes Treg and effector T cells

**Figure 3:**
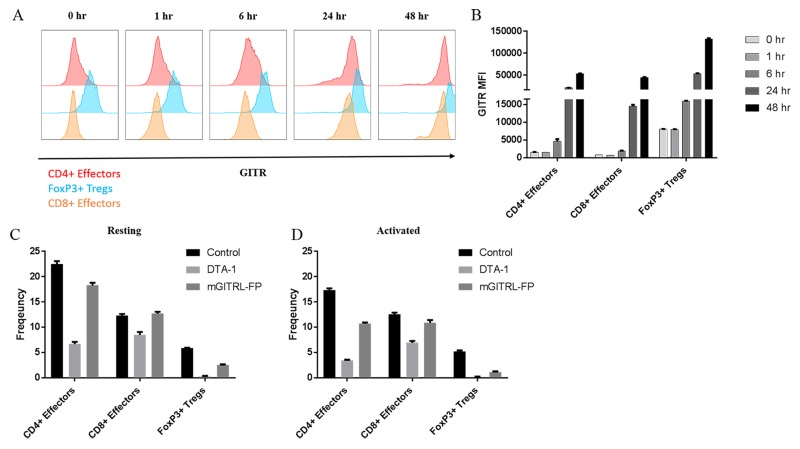
mGITRL-FP targets GITR-expressing T cells Splenocytes from C57BL/6 mice were stimulated *in vitro* by αCD3/CD28 and representative histograms **A.** and quantification of GITR mean fluorescence intensity (MFI) **B.** from activated T cells are shown. **C.** *Ex vivo* depletion of resting splenocytes (i.e., CD4^+^FoxP3^+^, CD4^+^FoxP3^-^, and CD8^+^ T cells) was determined in the presence of either DTA-1 or mGITRL-FP and complement. **D.** *Ex vivo* depletion of CD4^+^FoxP3^+^, CD4^+^FoxP3^-^, and CD8^+^ T cells determined in the presence of either DTA-1 or mGITRL-FP and complement and following activation with PMA + ionomycin. Bars in panels B-D indicate mean +/- SEM of quadruplicate wells.

Subsequent studies examined GITR expression levels on resting and activated T cells and their relative susceptibility for *in vitro* depletion by mGITRL-FP and αGITR mAb clone DTA-1. As previously reported [8, 9], GITR expression levels were much higher on resting CD4^+^FoxP3^+^ T cells when compared with resting CD4^+^FoxP3^-^ and CD8^+^ T cells (Figure 3A, 0 hours). *In vitro* activation of splenocytes with αCD3 and αCD28 mAbs significantly increased GITR expression levels on CD4^+^FoxP3^+^, CD4^+^FoxP3^-^ and CD8^+^ T cells (Figure 3A). Examining the relative MFI at 48 hours post-activation, GITR expression levels remained highest on CD4^+^FoxP3^+^ when compared with CD4^+^FoxP3^-^ and CD8^+^ T cells (Figure 3B). Others have demonstrated the ability of GITR targeting antibodies and antibody-like molecules to induce Fc-mediated depletion via ADCC [18, 19]; we further examined this utilizing a complement fixation/depletion approach. Incubation of resting splenocytes in the presence of mGITRL-FP and complement depleted more than 50% of CD4^+^FoxP3^+^ with minor reductions of CD4^+^FoxP3^-^ T cells (Figure 3C). For comparison, incubation with DTA-1 and complement reduced the frequencies of CD4^+^FoxP3^-^ and CD4^+^FoxP3^+^ T cells by 70-90%, while CD8^+^ T cells were reduced by approximately 30% (Figure 3C). As expected [18, 19], with activation and the accompanying increase in GITR expression was an increase in depletion [9], particularly in the CD4^+^FoxP3^-^ and CD4^+^FoxP3^+^ T cell subsets (Figure 3D). Incubation in the presence of mGITRL-FP and complement reduced the frequencies of the CD4^+^FoxP3^-^ and CD4^+^FoxP3^+^ T cell subsets by 40% and 80%, respectively, with no measurable reduction in CD8^+^ T cells. With the addition of the αGITR clone DTA-1 (positive control), 80-90% of CD4^+^FoxP3^+^ and CD4^+^FoxP3^-^ T cells were depleted, while reduction of CD8 ^+^ T cells was approximately 45% (Figure 3D).

### Temporal-dependent changes in T cell subsets following mGITRL-FP treatment alone or combined with rMVA/rF-CEA-TRICOM

**Figure 4:**
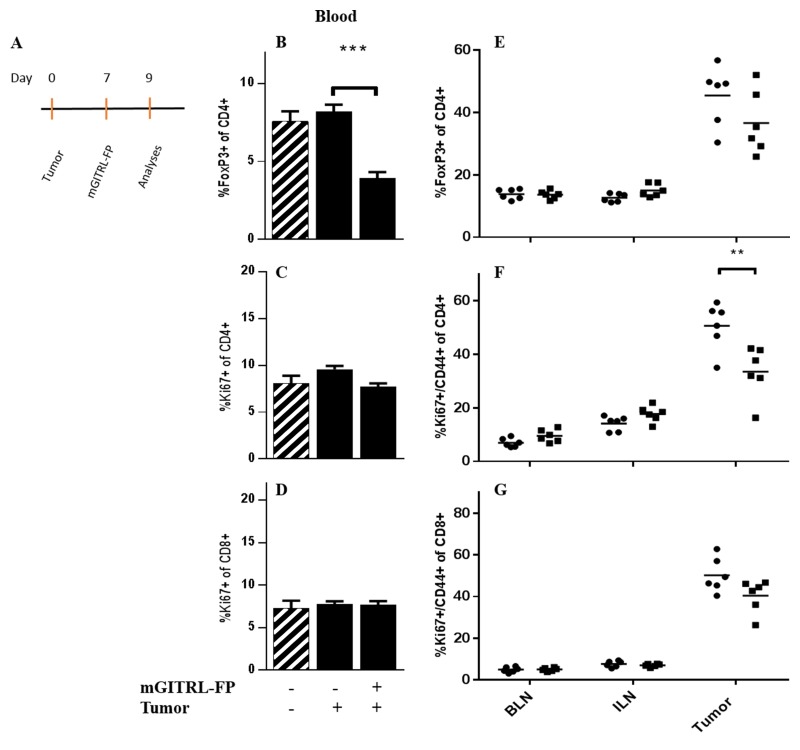
Effects of mGITRL-FP on the frequency of CD4^+^FoxP3^+^ and activated/proliferating effector T cells CEA.Tg mice bearing MC32A subcutaneous tumors were treated with 1.0 mg/kg mGITRL-FP 7 days post-tumor inoculation. Two days later **A.**, non-tumor bearing, untreated, and mGITRL-FP-treated MC32A tumor bearing CEA.Tg mice were euthanized (*n =* 5). Peripheral blood, brachial lymph nodes (BLN), inguinal lymph nodes (ILN) and tumors were collected. Cells were isolated and stained to identify different T cell subsets as well as activation and proliferative markers by flow cytometry. Bar graphs in panels **B**.-**D**. represent differences in circulating CD4^+^FoxP3^+^ (B), CD4^+^FoxP3^-^ (C), and CD8^+^ T cell (D) frequencies (*n* =5). Cross-hatched bars in panels B.-D. represent data from non-tumor bearing CEA.Tg mice (*n* =5). Error bars represent mean ± SEM. Panels **E.**-**G.** represent the changes in lymph node or tumor infiltrating CD4^+^FoxP3^+^ T cells (E), and CD4^+^FoxP3^-^ (F) or CD8^+^ (G) activated/proliferating effector T cells (*n* =6) from untreated (circles) and mGITRL-FP-treated (squares) CEA.Tg mice. ***P ≤ 0.001, **P ≤ 0.01 mGITRL-FP-treated vs. untreated CEA.Tg mice; Student’s t-test. Data are from a representative experiment that was repeated twice with similar results.

CEA.Tg mice received MC32A tumor cells and 7 days later received either a single, i.p. injection of mGITRL-FP (1.0 mg/kg) or a control antibody (Figure 1, Schedule C; Figure 4A). Forty-eight hours later, just prior to the priming vaccination, CEA.Tg mice from three treatment groups, MC32A‒tumor bearing CEA.Tg mice treated with mGITRL-FP or the control antibody and naïve CEA.Tg mice (no tumor inoculum), were examined for changes in the frequency of CD4^+^FoxP3^+^ T cells, and proliferative (i.e., Ki67^+^) CD4^+^FoxP3^-^ and CD8^+^ effector T cells in the peripheral blood (Figure 4B-4D). Also analyzed were non-tumor draining (brachial, BLN), tumor-draining lymph nodes (inguinal, ILN) and MC32A tumor microenvironment for changes in CD4^+^ FoxP3^+^ and proliferative/activated (i.e., Ki67^+^/CD44^+^) CD4^+^FoxP3^-^ and CD8^+^ effector T cells (Figure 4E-4G). In the peripheral blood of CEA.Tg mice bearing MC32A tumors, treated with mGITRL-FP, there was a significant decrease in the frequency of CD4^+^FoxP3^+^ (Figure 4B), not in the percentage of Ki67-expressing CD4^+^FoxP3^-^ or CD8^+^ effector T cells (Figure 4C and 4D). Within the tumor microenvironment of mGITRL-FP-treated CEA.Tg mice, a significant reduction in the percentage of Ki67^+^/CD44^+^ CD4^+^FoxP3^-^ T cells (Figure 4F) was found, while the frequencies of both CD4^+^FoxP3^+^ and Ki67^+^/CD44^+^ CD8^+^ T cells (Figure 4E and 4G) trended downward. Total intratumoral CD4^+^ and CD8^+^ T cell numbers in mGITRL-FP-treated CEA.Tg mice were not significantly different from control mice (data not shown), highlighting that depletion was specific to activated effectors, primarily Ki67^+^/CD44^+^ CD4^+^FoxP3^-^ T cells. These data were consistent with *in vitro* observations that demonstrated activation-induced upregulation of GITR, mGITRL-FP targeting, and subsequent increased effector T cell depletion (Figure 3). Within the ILN and BLN, no significant changes in the frequency/proliferation of those immune cell subsets from untreated or mGITRL-FP-treated CEA.Tg mice bearing MC32A tumors were observed (Figure 4E-4G).

**Figure 5:**
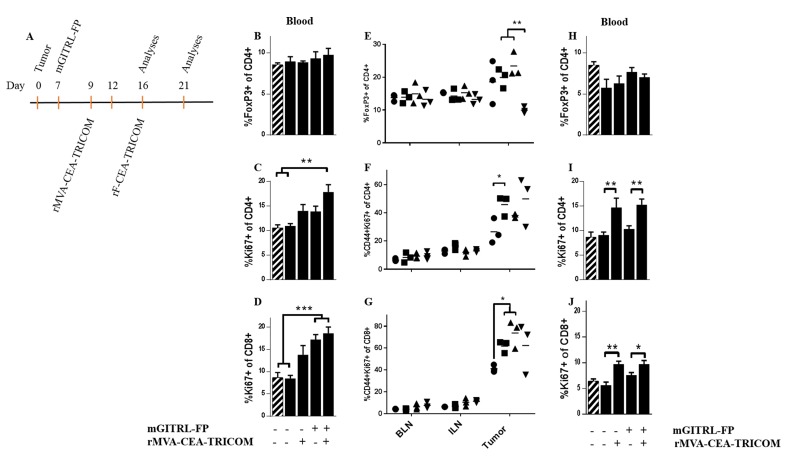
Combination rMVA/rF-CEA-TRICOM with mGITRL-FP increased T cell activation and proliferation in peripheral blood and reduced intratumoral CD4^+^FoxP3^+^ T cells **A**. CEA.Tg mice bearing MC32A s.c. tumors and treated with vaccine, GITRL-FP (1.0 mg/kg) alone or in combination were euthanized on either day 16 or 21 post-tumor inoculation. On day 16, untreated, vaccine, mGITRL-FP and vaccine with mGITRL-FP-treated CEA.Tg mice were analyzed for changes in circulating CD4^+^FoxP3^+^ T cell frequency (**B**.) and CD4^+^FoxP3^-^ (**C**. **P ≤ 0.01, vaccine + mGITRL-FP vs. untreated and naïve, non-tumor bearing CEA.Tg mice) and CD8^+^ (**D**. ***P ≤ 0.001, mGITRL-FP or vaccine + mGITRL-FP vs. untreated and naïve, non-tumor bearing CEA.Tg mice) T cell proliferation are shown (*n* = 5). Similar analyses were done on BLN, ILN and the tumor microenvironment of untreated (circles), vaccine (squares), mGITRL-FP (triangles) and vaccine with mGITRL-FP-treated (inverted triangles) mice: (**E**.) CD4^+^FoxP3^+^ T cell frequency, **P ≤ 0.01, vaccine or mGITRL-FP vs. vaccine + mGITRL-FP-treated CEA.Tg mice, (**F**.) CD44^+^Ki67^+^ CD4^+^FoxP3^-^, *P ≤ 0.01 and (**G**.) CD44^+^Ki67^+^ CD8^+^, *P ≤ 0.05, untreated vs. vaccine or mGITRL-FP-treated) (*n* = 3). On day 21 (**H**.-**J**.), changes in circulating CD4^+^FoxP3^+^ T cell frequency (H) and in CD4^+^FoxP3^-^ (I, **P ≤ 0.01, vaccine vs. untreated; vaccine + mGITRL-FP vs. mGITRL-FP alone) and CD8^+^ (J. *P ≤ 0.05, vaccine + mGITRL-FP vs. mGITRL-FP alone, **P ≤ 0.01, vaccine vs. untreated) T cell proliferation are shown (*n* = 5). Error bars (B-D; H-J) represent mean ± SEM, Student’s t-test. Cross-hatched bars (B-D; H-J) represent data from non-tumor bearing CEA.Tg mice (*n* = 5). Data are from a representative experiment that was repeated with similar results.

Untreated and mGITRL-FP-treated CEA.Tg mice bearing MC32A tumors were administered the priming vaccine on day 9 (Figure 5A) and 7 days later (day 16), peripheral blood (Figure 5B-5D), non-tumor draining (brachial, BLN), tumor-draining lymph nodes (inguinal, ILN) and tumors (Figure 5E-5G) were analyzed as described in Figure 4. Day 16 represents a breakpoint when MC32A tumor growth resumed in a majority of CEA.Tg mice treated with mGITRL-FP alone (Figure 2C), whereas antitumor efficacy was maintained with vaccine and mGITRL-FP treatment resulting in complete regression in 68% of CEA.Tg mice (Figure 1) and a significant improvement in survival (Figure 2G). On day 16, there were no differences in the frequencies of CD4^+^FoxP3^+^ T cells in the peripheral blood in any of the treatment groups of CEA.Tg mice (Figure 5B). However, Ki67^+^-expressing CD4^+^FoxP3^-^ T cells were significantly increased in the peripheral blood of CEA.Tg mice treated with the vaccine and mGITRL-FP (Figure 5C). Likewise, the percentage of Ki67^+^ CD8^+^ T cells was significantly increased in the peripheral blood of CEA.Tg mice treated with the vaccine and mGITRL-FP as well as mice that received mGITRL-FP alone (Figure 5D). The most profound differences in the immune cell subsets were found in the tumor microenvironment (Figure 5E-5G). Notably, the MC32A intratumoral frequency of CD4^+^FoxP3^+^ regulatory T cells was lowest (**P ≤ 0.01) in the CEA.Tg mice treated with the combined immunotherapeutics (Figure 5E). The frequency of CD4^+^FoxP3^+^ T cells in MC32A tumors from CEA.Tg mice treated with the vaccine and mGITRL-FP was <10% compared with frequencies of 15-25% in tumors from untreated, vaccine or mGITRL-FP-treated CEA.Tg mice (Figure 5E). CEA.Tg mice bearing MC32A tumors and treated with the vaccine alone had significantly higher (*P ≤ 0.05) percentages of Ki67^+^/CD44^+^ CD4^+^FoxP3^-^ T cells in the tumor microenvironment (Figure 5F). A similar increase was found for Ki67^+^/CD44^+^ CD8^+^ T cells in CEA.Tg mice treated with either the vaccine or mGITRL-FP as monotherapies (Figure 5G).

Additional analyses were done 12 days post priming vaccination and 5 days post boost (day 21, Figure 5H-5J). Frequencies of CD4^+^ FoxP3^+^ T cells in the peripheral blood were similar in CEA.Tg mice from all five groups (Figure 5H). The percentages of CD4^+^FoxP3^-^ and CD8^+^ T cells expressing Ki67^+^ in the peripheral blood were similar in control- and mGITRL-FP-treated CEA.Tg mice (Figure 5I and 5J). However, higher percentages of Ki67^+^-expressing CD4^+^FoxP3^-^ and CD8^+^ T cells were found in the peripheral blood from CEA.Tg mice that received the vaccine alone or combined with mGITRL-FP, suggesting the generation of long-lasting, durable effector T cell response in vaccinated CEA.Tg mice (Figure 5I and 5J). In conclusion, mGITRL-FP administered prior to the rMVA/rF-CEA-TRICOM vaccine elicits specific changes in immune cell subsets (i.e., reduction of CD4^+^FoxP3^+^ regulatory T cells/repopulation of T effectors) within the tumor microenvironment both of which support a robust and durable antitumor response. In addition, there were no overt signs of toxicity in mice in which mGITRL-FP alone or in combination with the rMVA/rF-CEA-TRICOM vaccine resulted in significant regression of the MC32A, CEA-expressing tumors.

### Immunofluorescent analysis of immune cell infiltrate into MC32A tumors

**Figure 6:**
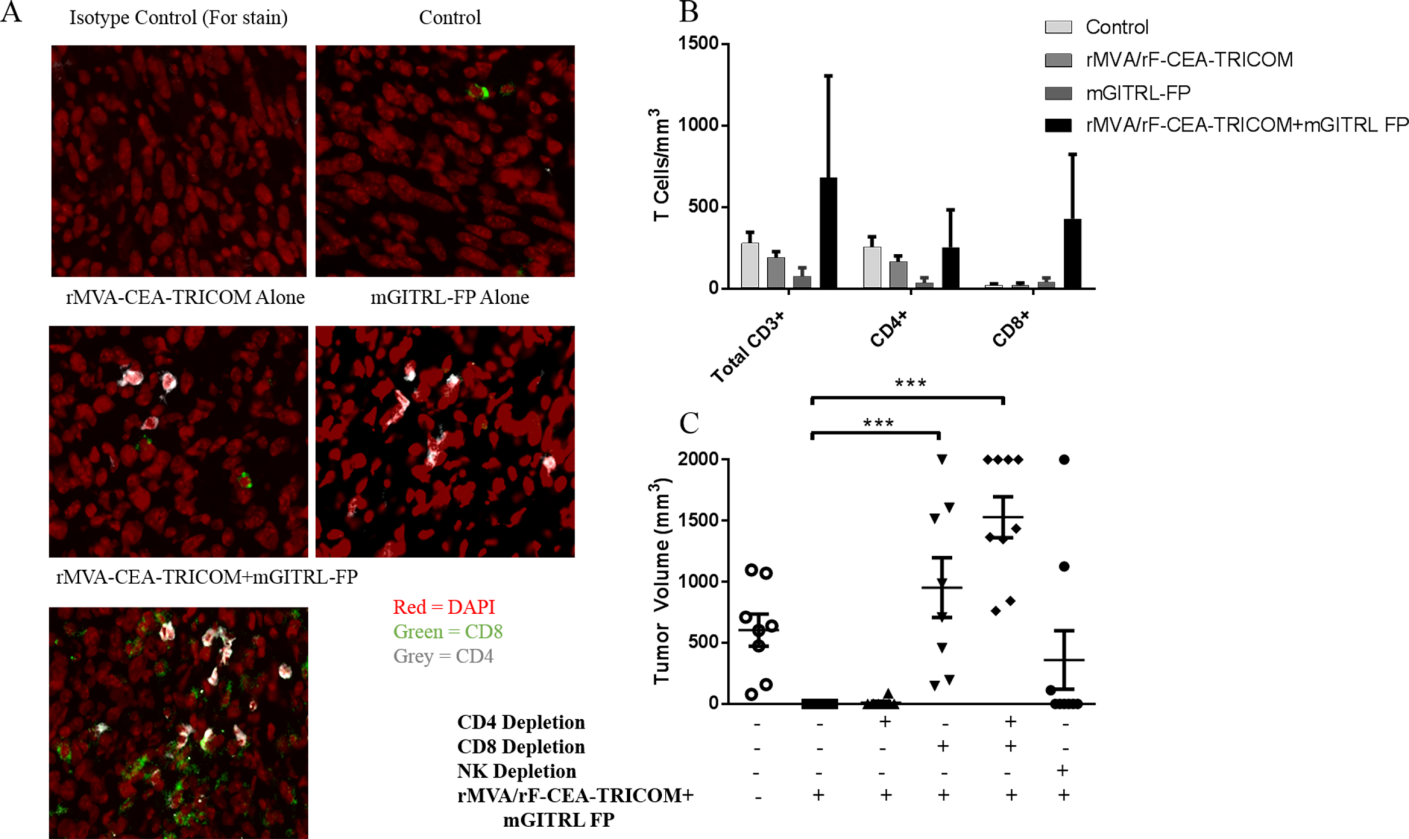
CD8^+^ T cells are required for anti-tumor efficacy resulting from combination rMVA/rF-CEA-TRICOM with mGITRL-FP treatment MC32A tumors were collected on day 16 (see Figure 5A) from CEA.Tg mice treated as outlined in Figures 4 and 5. **A**. CD4^+^ (grey) and CD8^+^ (green) T cells in the MC32A tumor microenvironment were visualized via confocal microscopy. **B.** Quantification of CD4^+^FoxP3^-^, CD8^+^ and CD3^+^ T cells in the MC32A tumor microenvironment from panel A. Cells were immunostained for T cell markers and T cell subsets were enumerated via flow cytometry and reported as the cell number/mm^3^ average tumor volume. Error bars represent mean ± SEM (*n* = 3). **C.** Roles of CD4^+^, CD8^+^, or natural killer (NK) cells in the anti-tumor response as determined by *in vivo* antibody depletions. Each data point represents a single mouse and the error bars represent mean ± SEM, Student’s t-test; ***P ≤ 0.001 (CD8^+^ or CD4^+^/8^+^ depleted vs. vaccine + mGITRL-FP-treated CEA.Tg mice).

Confirmation of tumor infiltrating effector T cells was performed via immunofluorescent (IF) staining and confocal analysis (Figure 6A). Enumeration of CD4^+^, CD8^+^ and CD3^+^ T cells in the tumor microenvironment confirmed an increase in CD8^+^ and CD3^+^ T cells in CEA.Tg mice treated with the vaccine and mGITRL-FP (Figure 6B). The number of CD4^+^, CD8^+^ and CD3^+^ T cells in the MC32A tumor microenvironment was lowest in CEA.Tg mice treated with mGITRL-FP alone (Figure 6B).

To determine the immune cell subset(s) involved in MC32A tumor rejection following vaccine + mGITRL-FP treatment, CEA.Tg mice were administered depleting antibodies for CD4, CD8, or NK cells (Figure 6C) and depletion was confirmed by flow cytometry (Supplementary Figures 1 and 2). CEA.Tg mice were inoculated with MC32A tumor cells 4 days post-initiation of depletion followed by the vaccine and mGITRL-FP as described for treatment Schedule C (Figures 1 and 2). It was interesting that CEA.Tg mice depleted of CD4^+^ T cells had no significant change in their ability to reject MC32A tumors (Figure 6C). The combination of the CD4^+^-depleting antibody and mGITRL-FP sustained depletion of regulatory T cells and when combined with mGITRL-FP agonist effects on CD8^+^ T cells, tumor regression occurred without the need for CD4^+^ T cells. NK cell depletion also led to no significant differences in tumor rejection, although two mice developed tumors whose growth kinetics were similar to that of the untreated mice. Tumor rejection following vaccine and mGITRL-FP treatment was completely lost in mice depleted of CD8^+^ T cells alone and when combined with CD4^+^ T cell depletion (Figure 6C).

### Enhanced immune memory in CEA.Tg mice treated with vaccine and mGITRL-FP

**Figure 7:**
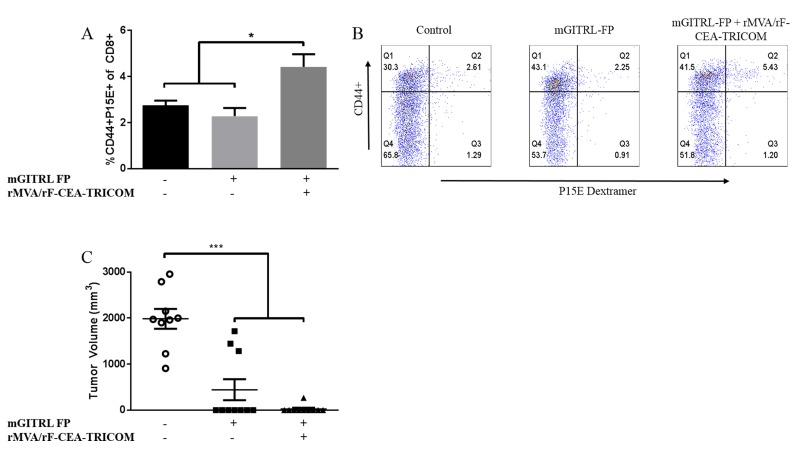
Increased memory formation following combination of rMVA/rF-CEA-TRICOM with mGITRL-FP CEA.Tg mice previously cured with either mGITRL-FP monotherapy (squares) or vaccine and mGITRL-FP combination therapy (triangles) were re-challenged with MC32A tumor cells. Naïve CEA.Tg mice also received a MC32A s.c. tumor challenge (circles). Six days post-tumor rechallenge, mice were bled and the frequency of TCR specificity for the CD8^+^ p15E rejection antigen determined via flow cytometry analysis **A.** (*n* = 5; *P ≤ 0.05, vaccine + mGITRL-FP vs. either untreated or mGITRL-FP treated CEA.Tg mice; Student’s t-test). Representative FACS plots demonstrate CD8^+^ T cell activation and p15E dextramer staining **B.** Tumor volume at 6 weeks post-rechallenge **C.** (*n* = 10; ***P ≤ 0.001, vaccine + mGITRL-FP or mGITRL-FP-treated vs. naïve untreated CEA.Tg mice; 2-way ANOVA). Horizontal bars represent the mean ± SEM.

T effector/memory cell formation was examined via dextramer staining of peripheral blood mononuclear cells (PBMCs) and flow cytometry analysis to determine the percentage of CD8^+^ T cells specific for the p15E rejection antigen. The p15E antigen is the transmembrane component of the retroviral envelope protein, is endogenous to MC32A tumors, and can act as a rejection antigen [20]. In fact, in a previous study [31], CD8^+^ immune response to the gp70, p15E, was far greater than those for CEA in CEA.Tg mice bearing MC32A tumors and immunized with the CEA-expressing recombinant poxvirus-based vaccines. The data showed that CEA was needed in the vaccine and tumor, but tumor regression was dependent on antigen cascade, particularly the presence of immunity to p15E. That antigen cascade was critical in the therapy of established murine tumors. A 2-fold increase in the percentage of activated peripheral blood CD8^+^ T cells specific for p15E was found in CEA.Tg mice that previously had rejected MC32A tumors following treatment with vaccine and mGITRL-FP versus either untreated or CEA.Tg mice treated with mGITRL-FP monotherapy (Figure 7A and 7B) (*n* = 5; *P* ≤ 0.05; Student’s t-test).

While 37% of mGITRL-FP alone and 68% of CEA.Tg mice treated with vaccine were cured (Figure 1) of MC32A tumors, it was of interest to determine the relative protective strength against subsequent challenge with MC32A tumor cells. CEA.Tg mice cured of their primary MC32A tumors received a re-challenge with MC32A subcutaneous tumors on the opposite flank. Both groups of mice cured of their primary MC32A tumors following treatment with mGITRL-FP alone or in combination with the vaccine rejected tumors upon rechallenge (*n* = 10; *P* ≤ 0.001; 2-way ANOVA) (Figure 7C). Mice that were previously treated with the combination immunotherapy trended towards a higher rate of protection from tumor rechallenge than mice previously treated with mGITR-FP alone. The findings support the hypothesis that T cell effector/memory develops in mGITRL-FP‒treated mice and can be expanded with the addition of vaccine.

## DISCUSSION

A previous study characterized mGITRL-FP along with its pharmacokinetic properties. Data supported the fusion protein’s ability to deplete/agonize GITR-expressing immune cells, which was a proposed mechanism for its actions as an immune therapeutic [30]. The present study was designed to investigate whether mGITRL-FP could function as an immune adjuvant when paired with rMVA/rF-CEA-TRICOM, a recombinant poxvirus-based vaccine. Treatment of CEA.Tg mice with the combination of vaccine and mGITRL-FP induced a robust antitumor immune response with regression of CEA-expressing tumors accompanied by durable tumor-free survival. Achieving those responses was strictly dependent on treatment schedule and the temporal relationship between mGITRL-FP administration and the rMVA-CEA-TRICOM priming vaccine was of paramount importance (Figures 1 and 2). Tumor regression was most sustainable (i.e., 68% complete tumor regression) when tumor bearing CEA.Tg mice were administered mGITRL-FP 2 days prior to the priming vaccine (Figure 1, Schedule C). When mGITRL-FP was administered during T cell activation/expansion, such as co-administration with the vaccine (Figure 1, Schedule A) or following tumor challenge of immunized mice (Figure 1, Schedule B), the resulting antitumor effects were unremarkable. One possible explanation is that the introduction of mGITRL-FP following vaccination could deplete vaccine-induced activated effectors.

The antitumor effects of mGITRL-FP when administered as a monotherapy has been attributed to depletion of intratumoral CD4^+^FoxP3^+^ regulatory T cells resulting in better CD4^+^FoxP3^-^ and CD8^+^:Treg ratios [30]. Those findings were the framework to examine changes in T cell subsets in the peripheral blood and the tumor microenvironment accompanying mGITRL-FP treatment prior to and during T cell priming with the rMVA-CEA-TRICOM vaccine (Figure 1, Schedule C). Approximately 90% mGITRL-FP drug clearance is predicted to occur *in vivo* within 2 days (day 9 on our treatment schedule) with complete clearance by 7 days [30]. A primary T cell response in regional draining lymph nodes is detectable for approximately 3 days following poxvirus immunization and continues for an additional 4-5 days [32]. Utilizing Schedule C, mGITRL-FP is able to perform its function (i.e., depletion and/or agonist activity), followed by clearance of the drug prior to the T cell response to vaccination. Analyses of the peripheral blood clearly showed a significant reduction in circulating CD4^+^FoxP3^+^ regulatory T cells. While within the MC32A tumors, mGITRL-FP treatment significantly reduces the number of proliferative/activated (i.e., Ki67^+^/CD44^+^) CD4^+^FoxP3^-^ effectors, with both CD4^+^FoxP3^+^ and Ki67^+^/CD44^+^ CD8^+^ trending downward. These data argue that in the mGITRL-FP-treated mice, at the time of the priming vaccination, the tumor microenvironment was more immuno-permissive by virtue of reduced numbers of (a) CD4^+^FoxP3^+^ (Figure 4E) and (b) proliferative CD4^+^FoxP3^-^ and CD8^+^ T cells (Figure 4F and 4G). The latter may provide “immune space” and allow repopulation by vaccine responding T cells within the tumor microenvironment [33].

From day 9 to day 16, the frequency of intratumoral proliferative/activated (i.e., Ki67^+^/CD44^+^) CD4^+^FoxP3^-^ and CD8^+^ T cells had returned to baseline levels, while that of CD4^+^FoxP3^+^ T cells continued to drop (Figure 5E). The resultant improvement in T_effector_:T_reg_ ratio appears to be a major contributor to the enhanced antitumor efficacy of the combined vaccine and mGITRL-FP treatment which coincides with previous findings [30]. In the peripheral blood of CEA.Tg mice treated with vaccine combined with mGITRL-FP, there was a significant increase in CD4^+^FoxP3^-^ and CD8^+^ T effectors (Figure 5C and 5D). One possible explanation is that GITR expression levels on circulating effector T cells may be sufficiently low that would allow those T cells to benefit from a proliferative burst from the agonist activity of the GITR–mGITRL-FP interaction while avoiding depletion. In contrast, elevated GITR levels on circulating and activated tumor infiltrating T_regs_ as well as activated effector T cells are sufficient for depletion. It seems that depletion following mGITRL-FP administration is more closely tied to relative GITR expression levels and GITR–mGITRL-FP interaction than to any specific T cell subset. Subsequent studies will examine the molecular events that underscore both the interactions of GITRL-FP with different immune cell subsets as well as with the accompanying reduction in tumor growth.

Two previous reports looked at the combination of different tumor vaccines with GITR ligation using the agonist DTA-1 antibody. In both cases, the anti-GITR antibody, DTA-1, was administered simultaneously during the priming stage with either a xenogeneic DNA [16] or bone marrow adherent cells pulsed with ovalbumin (OVA) vaccine [34]. Utilizing those schedules, DTA-1 addition enhanced tumor protection/regression and those effects were attributed to enhanced costimulation of CD4^+^FoxP3^-^ and CD8^+^ effector T cells with the simultaneous inhibition of T_reg_ suppression. While those findings seem to be in conflict with the present results, the differences might reside in the different molecules, DTA-1 versus mGITRL-FP, used to target GITR as well as the different vaccine platforms.

The rat IgG2b monoclonal antibody DTA-1 elicits agonist activity on T_regs_ and effector T cells [11, 12]. In addition, DTA-1 depletes intratumoral T_regs_ in an Fc-dependent manner [18, 19] and the depletory properties were specific for the tumor microenvironment, with no depletion occurring in the periphery [11]. Likewise, the mGITRL-FP exhibits agonist activity through native GITR-GITRL interactions, although any qualitative differences of agonist activity provided by DTA-1 and mGITRL-FP have not been elucidated. Like DTA-1, mGITRL-FP can deplete intratumoral T_regs_. Unlike DTA-1, mGITRL-FP also depletes circulating T_regs_ (Figure 4B) and supports the expansion of CD4^+^FoxP3^-^ and CD8^+^ effectors in the periphery (Figure 5C and 5D). With both DTA-1 and mGITRL-FP, a loss of intratumoral T_regs_ and effector T cells leaves the tumor microenvironment with a proclivity to be “reseeded” with immune cells. In the case of DTA-1, the tumor may be reseeded by circulating T_regs_ and effector T cells, which may explain the need for continued depletion via multiple DTA-1 treatments [34]. In contrast, reseeding after mGITRL-FP may occur primarily with effector T cells, due to reduced T_regs_ and increased effectors in the periphery, eliminating the need for multiple mGITRL-FP treatments. When combined with vaccine, there is an increased likelihood of reseeding with tumor specific effectors expanded via vaccination. It remains possible that mGITRL-FP-induced depletion after vaccination may deplete vaccine-induced/expanded effector T cells, which may explain the reduced efficacy of combination treatment seen in Schedule A (Figure 1).

With regard to a viral-based vaccine, such as rMVA-CEA-TRICOM, its persistence at the injection site over multiple days might be expected to be a more potent priming vaccine than either the DNA or OVA peptide-pulsed dendritic cell vaccines. The relative strengths of the different vaccine platforms would also dictate the depth/duration of the proinflammatory events at the injection site which, in turn, can lead to GITR upregulation of activated immune cell subsets at the regional draining lymph node. It becomes possible that the different effects of DTA-1 and mGITRL-FP could be due to the strength of the vaccines and, more importantly, the extent of GITR upregulation on immune cells. Couple those vagaries with the possibility that combining a vaccine designed to target a self-antigen, similar to what is seen in our MC32A model, and GITR ligation may also increase the possibility of autoimmunity. Future studies should include a careful monitoring of any toxicity that involves tissues that constitutively express the target antigen [35]. Then preclinical studies that combine GITR ligation with different vaccine platforms become an even more important step prior to the testing of a combination in early clinical trials. Nonetheless, targeting GITR with mGITRL-FP prior to treatment with a therapeutic poxvirus-based antigen-specific vaccine acts as a powerful immune adjuvant, resulting in a significant improvement in tumor regression, durable tumor-free survival and the generation of protective immune memory.

## MATERIALS AND METHODS

### Murine models

Adult female C57BL/6 mice were purchased from Charles River Laboratories (Frederick, MD). Mice that express the human *CEA* gene on the C57BL/6 background were kindly provided by Dr. John Shively (City of Hope, Duarte, CA). Complete description of the generation of the CEA.Tg mice has been published [25]. Briefly, a cosmid clone cosCEAl containing the complete coding region of the human CEA gene, including 32.6kb of the 5’-flanking and 5 kb of the 3’-flanking regions, was used to generate the CEA.Tg mice. CEA protein expression was similar to that found in humans, predominately in the gastrointestinal tract, whereas other sites, such the trachea, esophagus, small intestine, and lung, also expressed CEA. CEA expression has been reported in murine thymic epithelial cells (mTEC) of CEA.Tg mice similar to expression levels in human TEC. In the CEA.Tg mouse, CEA expression in the mTEC resulted in tolerization of a major fraction of the T cell repertoire [36]. All animals were housed and maintained under pathogen-free conditions in microisolator cages, and were 2–6 months old at the start of each study. Animal care was in compliance with the recommendations of *The Guide for Care and Use of Laboratory Animals* (National Research Council).

### Subcutaneous tumor growth studies

MC32A, a murine colorectal tumor cell line expressing human *CEA* and developed in the Laboratory of Tumor Immunology and Biology, Center for Cancer Research, National Cancer Institute, National Institutes of Health, Bethesda, MD [37], was routinely grown *in vitro* in DMEM (Cellgro/Mediatech, Manassas, VA) containing 10% fetal bovine serum (FBS), 0.1mM non-essential amino acids (NEAA), 1mM sodium pyruvate, 2mM L-glutamine, 50μg/ml gentamicin, 10mM HEPES, and penicillin/streptomycin. MC32A cells were subjected to PCR based MTBM testing and determined to be negative for Mycoplasma and other murine viral and bacterial pathogens. Cells were injected within 10 passages from the time of thawing and were routinely confirmed to express the *CEA* transgene. To establish a subcutaneous tumor model, MC32A (3x10^5^) cells were injected s.c. into the right rear flank of adult mice. One to 2 weeks later, when the average tumor volume was 40-60 mm^3^, mice were randomized and treatment was initiated. Tumors were measured twice weekly using calipers, and the tumor volume was calculated as: Volume = 0.5 x (width)^2^ x (length). For tumor re-challenge studies, 3x10^5^ MC32A cells were injected into the opposite flank of mice previously cured of their primary tumor.

### Treatments

A murine GITR ligand fusion protein (mGITRL-FP) was constructed and produced by MedImmune LLC (Gaithersburg, MD) under a Cooperative Research and Development Agreement (CRADA). The molecule consisted, from N- and C-terminus, of a fragment crystallisable (Fc) region of an immunoglobulin G (IgG), the yeast-derived coiled coil GCN4 pII and the extracellular (GITR-binding) domain (ECD) of murine GITR ligand (mGITRL-FP). Both mGITRL-FP mIgG2a and an anti-GITR (DTA-1) induced NF-kB reporter gene cell assay [30]. CEA.Tg mice were administered either 1.0 or 0.1 mg/kg body weight via intraperitoneal injection at the designated time points.

Recombinant poxvirus-based vaccines that were used included Modified Vaccinia virus Ankara (rMVA-) and fowlpox (rF-) engineered to express genes encoding human *CEA* and three murine costimulatory molecules, *B7.1*, *ICAM-1* and *LFA-3* (designated TRICOM). Those vaccines are termed rMVA- or rF-CEA-TRICOM. Details of the construction, production and efficacy of incorporating the costimulatory in the recombinant poxvirus-based vaccines have been published [20, 21]. All vaccines were administered s.c. at a dose of 10^8^ pfu in 100 µl HBSS on the rear flank opposite from the tumor cell injection.

### *In vivo* immune cell subset depletions

For *in vivo* depletion studies, mice were administered four daily i.p injections of depleting antibodies prior to tumor instillation, followed by weekly i.p injections of depleting agents after initiation of tumor. CD4^+^ T cell depletion was accomplished via administration of 100 µg/injection of anti-CD4 clone GK1.5 (BioXcell, Branford, CT). CD8^+^ T cell depletion was accomplished via administration of 100 μg/injection of anti-CD8 clone 2.43 (BioXcell, Branford, CT). NK cell depletion was accomplished via administration of 25 µL/injection of anti-NK1.1 clone PK136 (BioXcell, Branford, CT) and 25 µg/injection of polyclonal anti-asialo GM1 (Cedarlane Laboratories, Burlington, Canada).

### *In vitro* T cell stimulation/depletion assays

Splenocytes were cultured in RPMI (Cellgro/Mediatech) supplemented with 10% FBS at a concentration of 5x10^5^ cells per well in a 96-well round-bottom plate. *In vitro* stimulation was performed by adding αCD3 (clone 145-2C11) and αCD28 (clone 37.51) (eBioscience, San Diego, CA) in solution to a final concentration of 1 µg/ml and 2 µg/ml, respectively, or utilizing Cell Stimulation cocktail (PMA/Ionomycin) diluted 1:500 (eBioscience). Fc-mediated depletion was investigated by culturing and treating cells as described, followed by the addition of 25 µL of rabbit complement (Cedarlane Laboratories) for the final 30 minutes of culture at 37^°^C. At the end of the culture period, cells were washed in FACS buffer prior to fixation using FoxP3/transcription factor kit (eBioscience).

### Flow cytometry

Antibodies used for flow cytometry or immunofluorescence were purchased from BD Biosciences (San Jose, CA), eBioscience, or BioLegend (San Diego, CA). Fluorescently conjugated antibodies specific for CD3 (145-2C11), CD4 (RM4-5), CD8 (53-6.7), CD25 (PC61), FoxP3 (FJK-16s), GITR (DTA-1), CD62L (MEL-14), CD44 (IM7), Ki67 (B56) were used for flow cytometry. Dextramer specific for H2K^b^/P15E was purchased from Immudex (Copenhagen, Denmark).

Prior to flow cytometric analyses, single cell suspensions were prepared from spleens, lymph nodes, or tumors using mechanical dissociation. Red blood cell lysis was performed with ACK buffer (Quality Biologicals, Inc., Gaithersburg, MD), and single cell suspensions were prepared by filtering through a 40 µm nylon cell strainer. PBMCs were isolated from blood utilizing Lympholyte-M (Cedarlane Laboratories) per the manufacturer’s specifications. Cell suspensions were stained on ice with antibodies diluted in FACS buffer. Dead cells were stained and excluded via Live/Dead fixable stain (Life Technologies, Carlsbad, CA). Intracellular staining was performed using FoxP3/transcription factor kit (eBioscience), according to the manufacturer’s instructions. When necessary, cells were enumerated utilizing AccuCheck Counting Beads (Invitrogen, Carlsbad, CA) per the manufacturer’s instructions. Where applicable, cells were fixed using 1.5% microscopy grade paraformaldehyde (PFA, Electron Microscopy Sciences, Hatfield, PA) diluted in FACS buffer. Cytometry data was acquired via 3 laser FACSVERSE or 5 laser Fortessa (BD Biosciences). Data was analyzed via FlowJo (FlowJo, LLC, Ashland, OR).

### Confocal microscopy

Tumors were flash frozen in O.C.T. Compound (Tissue-Tek, Torrance, CA) and sectioned by HistoServ, Inc. (Germantown, MD). Sections were thawed in 4% microscopy grade PFA (Electron Microscopy Sciences) diluted in PBS for 15 minutes at room temperature (RT). Sections were permeabilized using 0.5% Triton X100 diluted in PBS for 10 minutes at RT and following washes were incubated for 1 hour at RT in blocking buffer: 1:1 dilution of Superblock (Thermo Fisher Scientific, Waltham, MA) in PBS with 1:100 Fc block (clone 2.4G2, BD Biosciences). Antibodies used for immunofluorescence were purchased from eBioscience. Alexa Fluor conjugated antibodies specific for CD4 (GK1.5) and CD8 (53-6.7) were diluted in blocking buffer. Sections were mounted using Prolong Gold Antifade Reagent with or without DAPI (Life Technologies). Sections were imaged using a Zeiss LSM 880 NLM Airyscan confocal microscope and analyzed using ZEN lite software (Zeiss, Thornwood, NY).

## SUPPLEMENTARY MATERIALS FIGURES



## Abbreviations

BLN: brachial lymph node
CEA: carcinoembryonic antigen
CEA.Tg: CEA transgenic
DCs: dendritic cells
Fc: fragment crystallisable
GITR: glucocorticoid-induced tumor necrosis factor receptor
IF: immunofluorescent
IgG: immunoglobulin G
ILN: inguinal lymph node
MFI: mean fluorescence intensity
GITRL-FP: murine GITR ligand fusion protein
mTEC: murine thymic epithelial cells
NF-kB: nuclear factor kB
NK: natural killer
OVA: ovalbumin
PBMCs: peripheral blood mononuclear cells
PFA: paraformaldehyde
rF: recombinant fowlpox
rMVA: recombinant Modified Vaccinia virus Ankara
TCR: T cell receptor
TNF: tumor necrosis factor
TNFRSF18: tumor necrosis factor receptor superfamily member 18
T_reg_: regulatory T cells

## References

[1] Immunotherapy Agent Workshop NCI. July 12, 2007 https://ncifrederick.cancer.gov/research/brb/workshops/nci%20immunotherapy%20workshop%207-12-07.pdf.

[2] Kwon B, Yu KY, Ni J, Yu GL, Jang IK, Kim YJ, Xing L, Liu D, Wang SX, Kwon BS (1999). Identification of a novel activation-inducible protein of the tumor necrosis factor receptor superfamily and its ligand. J Biol Chem.

[3] Nocentini G, Giunchi L, Ronchetti S, Krausz LT, Bartoli A, Moraca R, Migliorati G, Riccardi C (1997). A new member of the tumor necrosis factor/nerve growth factor receptor family inhibits T cell receptor-induced apoptosis. Proc Natl Acad Sci U S A.

[4] Schaer DA, Hirschhorn-Cymerman D, Wolchok JD (2014). Targeting tumor-necrosis factor receptor pathways for tumor immunotherapy. J Immunother Cancer.

[5] Nocentini G, Ronchetti S, Petrillo MG, Riccardi C (2012). Pharmacological modulation of GITRL/GITR system: therapeutic perspectives. Br J Pharmacol.

[6] Schaer DA, Murphy JT, Wolchok JD (2012). Modulation of GITR for cancer immunotherapy. Curr Opin Immunol.

[7] Shimizu J, Yamazaki S, Takahashi T, Ishida Y, Sakaguchi S (2002). Stimulation of CD25(+)CD4(+) regulatory T cells through GITR breaks immunological self-tolerance. Nat Immunol.

[8] Esparza EM, Arch RH (2005). Glucocorticoid-induced TNF receptor, a costimulatory receptor on naive and activated T cells, uses TNF receptor-associated factor 2 in a novel fashion as an inhibitor of NF-kappa B activation. J Immunol.

[9] Esparza EM, Arch RH (2005). Glucocorticoid-induced TNF receptor functions as a costimulatory receptor that promotes survival in early phases of T cell activation. J Immunol.

[10] Stephens GL, McHugh RS, Whitters MJ, Young DA, Luxenberg D, Carreno BM, Collins M, Shevach EM (2004). Engagement of glucocorticoid-induced TNFR family-related receptor on effector T cells by its ligand mediates resistance to suppression by CD4+CD25+ T cells. J Immunol.

[11] Cohen AD, Schaer DA, Liu C, Li Y, Hirschhorn-Cymmerman D, Kim SC, Diab A, Rizzuto G, Duan F, Perales MA, Merghoub T, Houghton AN, Wolchok JD (2010). Agonist anti-GITR monoclonal antibody induces melanoma tumor immunity in mice by altering regulatory T cell stability and intra-tumor accumulation. PLoS One.

[12] Ko K, Yamazaki S, Nakamura K, Nishioka T, Hirota K, Yamaguchi T, Shimizu J, Nomura T, Chiba T, Sakaguchi S (2005). Treatment of advanced tumors with agonistic anti-GITR mAb and its effects on tumor-infiltrating Foxp3+CD25+CD4+ regulatory T cells. J Exp Med.

[13] Piao J, Kamimura Y, Iwai H, Cao Y, Kikuchi K, Hashiguchi M, Masunaga T, Jiang H, Tamura K, Sakaguchi S, Azuma M (2009). Enhancement of T-cell-mediated anti-tumour immunity via the ectopically expressed glucocorticoid-induced tumour necrosis factor receptor-related receptor ligand (GITRL) on tumours. Immunology.

[14] Turk MJ, Guevara-Patino JA, Rizzuto GA, Engelhorn ME, Sakaguchi S, Houghton AN (2004). Concomitant tumor immunity to a poorly immunogenic melanoma is prevented by regulatory T cells. J Exp Med.

[15] Schaer DA, Budhu S, Liu C, Bryson C, Malandro N, Cohen A, Zhong H, Yang X, Houghton AN, Merghoub T, Wolchok JD (2013). GITR pathway activation abrogates tumor immune suppression through loss of regulatory T cell lineage stability. Cancer Immunol Res.

[16] Cohen AD, Diab A, Perales MA, Wolchok JD, Rizzuto G, Merghoub T, Huggins D, Liu C, Turk MJ, Restifo NP, Sakaguchi S, Houghton AN (2006). Agonist anti-GITR antibody enhances vaccine-induced CD8(+) T-cell responses and tumor immunity. Cancer Res.

[17] Nishikawa H, Sakaguchi S (2010). Regulatory T cells in tumor immunity. Int J Cancer.

[18] Bulliard Y, Jolicoeur R, Windman M, Rue SM, Ettenberg S, Knee DA, Wilson NS, Dranoff G, Brogdon JL (2013). Activating Fc gamma receptors contribute to the antitumor activities of immunoregulatory receptor-targeting antibodies. J Exp Med.

[19] Mahne A, Mauze S, Joyce-Shaikh B, Xia J, Bowman E, Beebe A, Cua D, Jain R (2017). Dual roles for regulatory T cell depletion and co-stimulatory signaling in agonistic GITR targeting for tumor immunotherapy. Cancer Res.

[20] Hodge JW, Sabzevari H, Yafal AG, Gritz L, Lorenz MG, Schlom J (1999). A triad of costimulatory molecules synergize to amplify T-cell activation. Cancer Res.

[21] Hodge JW, Chakraborty M, Kudo-Saito C, Garnett CT, Schlom J (2005). Multiple costimulatory modalities enhance CTL avidity. J Immunol.

[22] Gold P, Freedman SO (1965). Demonstration of tumor-specific antigens in human colonic carcinomata by immunological tolerance and absorption techniques. J Exp Med.

[23] Guadagni F, Roselli M, Cosimelli M, Spila A, Cavaliere F, Arcuri R, D’Alessandro R, Fracasso PL, Casale V, Vecchione A, Casciani CU, Greiner JW, Schlom J (1997). Quantitative analysis of CEA expression in colorectal adenocarcinoma and serum: lack of correlation. Int J Cancer.

[24] Thompson JA, Grunert F, Zimmermann W (1991). Carcinoembryonic antigen gene family: molecular biology and clinical perspectives. J Clin Lab Anal.

[25] Clarke P, Mann J, Simpson JF, Rickard-Dickson K, Primus FJ (1998). Mice transgenic for human carcinoembryonic antigen as a model for immunotherapy. Cancer Res.

[26] Greiner JW, Zeytin H, Anver MR, Schlom J (2002). Vaccine-based therapy directed against carcinoembryonic antigen demonstrates antitumor activity on spontaneous intestinal tumors in the absence of autoimmunity. Cancer Res.

[27] Kass E, Panicali DL, Mazzara G, Schlom J, Greiner JW (2001). Granulocyte/macrophage-colony stimulating factor produced by recombinant avian poxviruses enriches the regional lymph nodes with antigen-presenting cells and acts as an immunoadjuvant. Cancer Res.

[28] Kass E, Schlom J, Thompson J, Guadagni F, Graziano P, Greiner JW (1999). Induction of protective host immunity to carcinoembryonic antigen (CEA), a self-antigen in CEA transgenic mice, by immunizing with a recombinant vaccinia-CEA virus. Cancer Res.

[29] Mizobata S, Tompkins K, Simpson JF, Shyr Y, Primus FJ (2000). Induction of cytotoxic T cells and their antitumor activity in mice transgenic for carcinoembryonic antigen. Cancer Immunol Immunother.

[30] Leyland R, Watkins A, Mulgrew K, Holoweckyj N, Bamber L, Tigue NJ, Offer E, Andrews J, Yan L, Mullins S, Oberst MD, Coates Ulrichsen J, Leinster DA (2017). A novel murine GITR ligand fusion protein induces antitumor activity as a monotherapy, which is further enhanced in combination with an OX40 agonist. Clin Cancer Res.

[31] Kudo-Saito C, Schlom J, Hodge JW (2005). Induction of an antigen cascade by diversified subcutaneous/intratumoral vaccination is associated with antitumor responses. Clin Cancer Res.

[32] Volz A, Sutter G (2017). Modified Vaccinia Virus Ankara: history, value in basic research, and current perspectives for vaccine development. Adv Virus Res.

[33] Stockinger B, Barthlott T, Kassiotis G (2004). The concept of space and competition in immune regulation. Immunology.

[34] Zhu LX, Davoodi M, Srivastava MK, Kachroo P, Lee JM (2015). St John M, Harris-White M, Huang M, Strieter RM, Dubinett S, Sharma S. GITR agonist enhances vaccination responses in lung cancer. OncoImmunology.

[35] Kohm AP, Williams JS, Miller SD (2004). Cutting edge: ligation of the glucocorticoid-induced TNF receptor enhances autoreactive CD4+ T cell activation and experimental autoimmune encephalomyelitis. J Immunol.

[36] Bos R, van Duikeren S, van Hall T, Kaaijk P, Taubert R, Kyewski B, Klein L, Melief CJ, Offringa R (2005). Expression of a natural tumor antigen by thymic epithelial cells impairs the tumor-protective CD4+ T-cell repertoire. Cancer Res.

[37] Robbins PF, Kantor JA, Salgaller M, Hand PH, Fernsten PD, Schlom J (1991). Transduction and expression of the human carcinoembryonic antigen gene in a murine colon carcinoma cell line. Cancer Res.

