# Supplementary material for: Enhanced immunotherapy by combining a vaccine with a novel murine GITR ligand fusion protein
Source: Oncotarget. 2017 Sep 7;8(43):73469–82. doi: 10.18632/oncotarget.20703 (PMC5650275; doi:10.18632/oncotarget.20703)
Supplement: Supplementary file 1 [file oncotarget-08-73469-s001.pdf]

Enhanced immunotherapy by combining a vaccine with a novel murine GITR ligand fusion protein

SUPPLEMENTARY MATERIALS

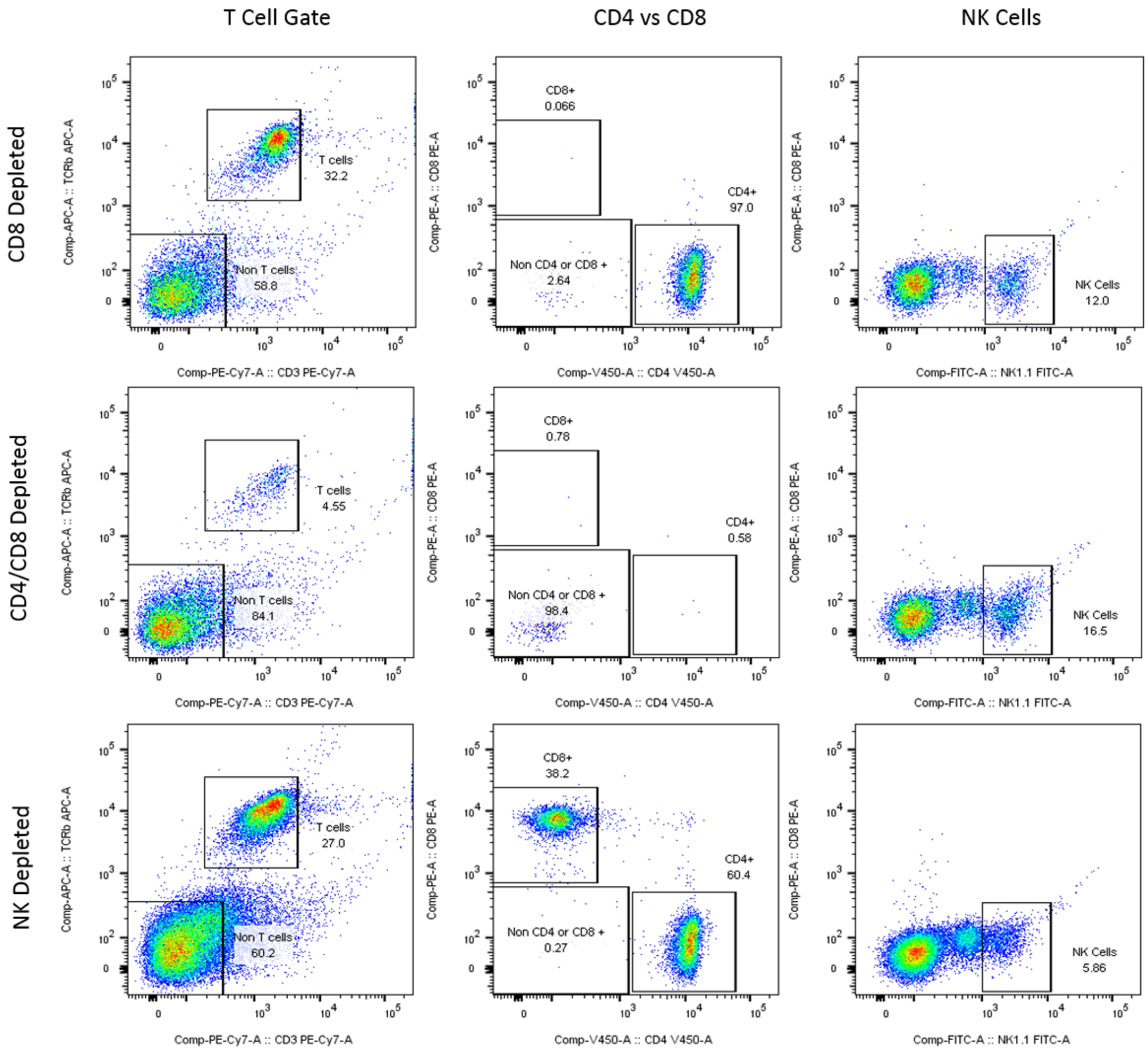

Supplementary Figure S1. Confirmation of subset depletion: CEA expressing C57BL/6 mice were administered depleting antibodies specific for the designated immune subset. Peripheral blood was stained for flow cytometry analysis to confirm depletion.

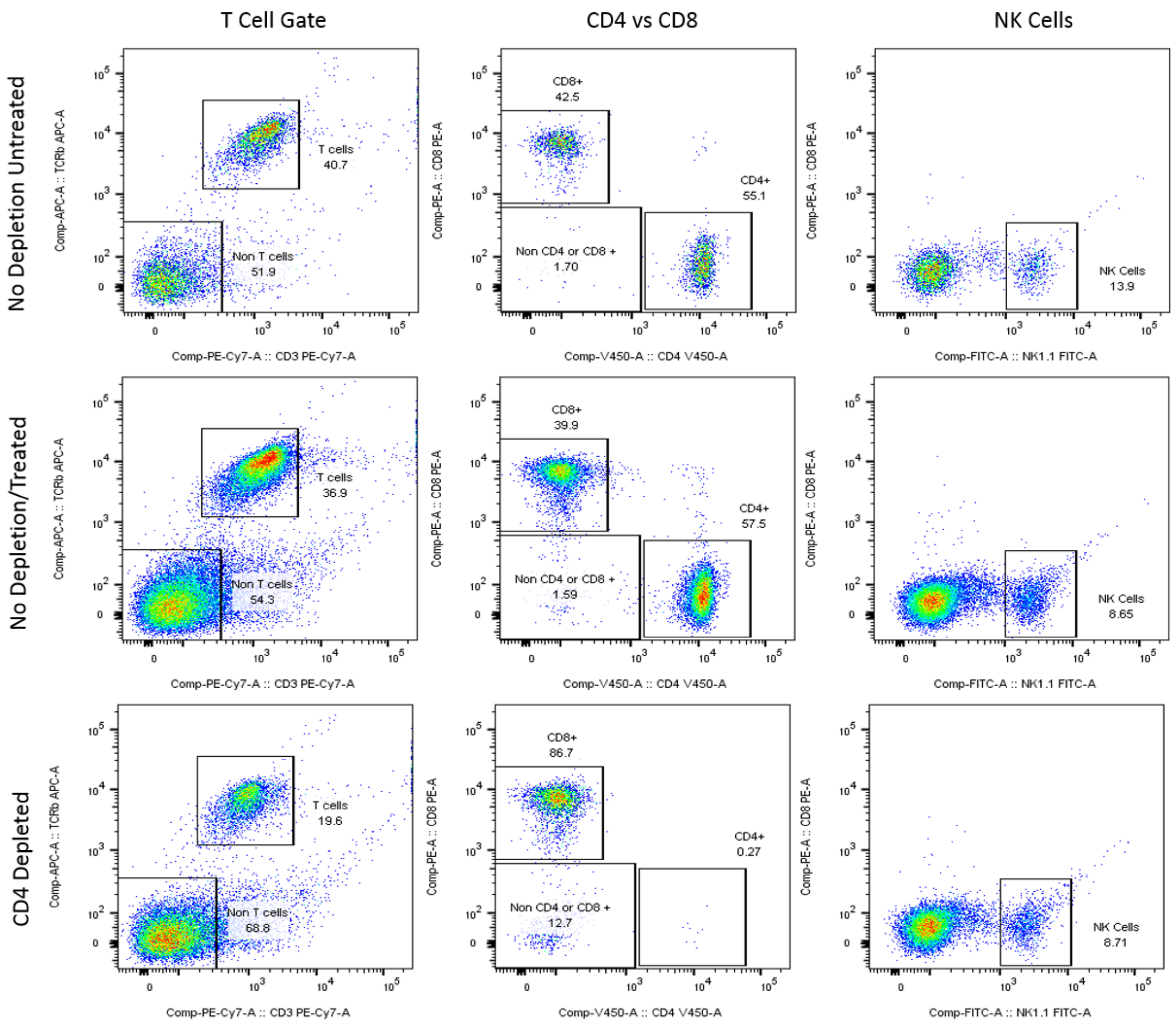

Supplementary Figure S2. Confirmation of subset depletion: CEA expressing C57BL/6 mice were administered depleting antibodies specific for the designated immune subset. Peripheral blood was stained for flow cytometry analysis to confirm depletion.
